# Study on carbapenemase-producing bacteria by matrix-assisted laser desorption/ionization approach

**DOI:** 10.1371/journal.pone.0247369

**Published:** 2021-03-18

**Authors:** Michał Złoch, Paweł Pomastowski, Markus Peer, Katrin Sparbier, Markus Kostrzewa, Bogusław Buszewski

**Affiliations:** 1 Centre for Modern Interdisciplinary Technologies, Nicolaus Copernicus University, Toruń, Poland; 2 Bruker Daltonik GmbH, Bremen, Germany; 3 Chair of Environmental Chemistry and Bioanalytics, Faculty of Chemistry, Nicolaus Copernicus University, Toruń, Poland; Universita degli Studi di Parma, ITALY

## Abstract

The development of new techniques for the detection of carbapenemase activity is of great importance since the increased incident of resistance against carbapenems represents a serious threat to global public health. In this context, the **matrix-assisted laser desorption/ionization approach** already demonstrated to be a reliable tool for rapid carbapenemase detection. As a newly developed test, there is still a lack of in-depth analysis of its robustness and possible wider application. The main goal of this study was to evaluate the potential for using the design MBT STAR-Carba assay as the pre-characterization method for *Enterobacterales* and *P*. *aeruginosa* strains in terms of the produced classes of carbapenemases using modified procedure parameters—various suspension densities and incubation times. Moreover, its usefulness for the in-depth analysis and characterization of metallo-β-lactamases (MBL) was tested by applying inhibition assays. In this study, the designed assay proved to be a sensitive tool for the detection of carbapenemase hydrolytic activity, which can be successfully used to partially classify the class of carbapenemase present. Additionally, the use of defined high concentration suspensions would allow to shorten the incubation time to 1 minute for certain strains. Considering that the assay was also suitable to investigate the effect of different inhibitors on the MBL activity, it demonstrates far higher discriminatory potential than only a rapid routine carbapenemase detection tool and could be used as a susceptibility assay.

## Introduction

The increasing prevalence of drug-resistant bacteria is a serious threat to human health and life around the world. Carbapenems are amongst the latest developed β-lactam antibiotics still possessing a broad spectrum of bactericidal activity, therefore often used as last resort medication for the treatment of severe bacterial infections [1–3]. Therefore, the global rise of carbapenemase-producing bacteria (CPB) incidence represents an increasing threat to the healthcare system and patient safety and is of great concern worldwide [4,5]. Reported increase in resistance to carbapenems among *Enterobacterales* as well as *Pseudomonas aeruginosa* isolates is linked to their high consumption during treatment of infections caused by extended-spectrum-β-lactamases (ESBLs) harboring microorganisms [6,7].

Although carbapenem resistance can occur in several ways, enzymatic hydrolysis of an antibiotic (carbapenemase expression) is considered of more clinical concern since CPB demonstrate the greatest potential for rapid dissemination within hospital settings via horizontal transfer of the carbapenemase-encoding genes as well as clonal expansion [8,9]. Regarding Ambler classification, carbapenemases are classified into 3 main groups–class A serine β-lactamases mostly represented by KPC (*Klebsiella pneumoniae* carbapenemase), class B metallo-β-lactamases–MBL (e.g. NDM-, VIM-, IMP-, and GIM-types) as well as class D, another serine β-lactamase class, including OXA-48 group like OXA-48, OXA-162, or OXA-181 [10].

Detection of carbapenemases is still challenging for clinicians since interpretative criteria recommended by the EUCAST or the Clinical and Laboratory Standards Institute (CLSI) lead to nearly 20% of false negative results in classic antibiotic susceptibility testing using breakpoint values for carbapenems even with low screening cut-offs [1,10]. Therefore, various phenotypic tests for detection of carbapenemase producers have been developed, e.g. Carbapenem Inactivation Method, gradient MIC strips (Etest) or combined disc inhibitory tests for carbapenemase activity [11–13]. Although growth-based phenotypic tests are inexpensive and easy to perform, they, nevertheless, require additional incubation thus significantly extending the time needed to receive results to 24–72 hours and very often are characterized by lack of sensitivity and specificity [10,14]. These drawbacks could be overcome using molecular methods such as targeted PCR assays receiving results within a few hours (<4 h); however, with higher costs, the need for significant expertise along with the limitation to a predefined range of known carbapenem-resistance genes [3,13].

In view of the mentioned drawbacks, rapid biochemical assays based on carbapenem-hydrolyzing activity detection are considered as the most powerful methods which facilitate the indication of the presence of all classes and variants of carbapenemases, including the rarest ones, with high specificity and sensitivity [15,16]. Recently, special attention has been paid to the use of matrix assisted laser desorption ionization-time of flight mass spectrometry (MALDI-TOF MS), as an approach that shortens the time-to-result for antibiotic susceptibility testing [17]. This technique proved to produce also reliable same-day results for detecting β-lactamase activity [18,19]. So far, several publications have demonstrated the feasibility of MALDI-TOF MS analysis to detect carbapenemase activity [20–23]. They relate to the data analysis performed based on the manual calculations and self-developed algorithms for peak patterns investigation, thus difficult to implement in routine workflows.

Currently, MBT STAR-Carba assay is extensively used as a fast qualitative test of the carbapenemases presence. Therefore, the main goal of this study was to evaluate the potential for using the design MBT STAR-Carba assay also as the pre-characterization method of the bacterial strains in terms of the produced classes of carbapenemases using modified procedure parameters, here: different bacterial suspension densities and various incubation times. In total twelve (12) different *Enterobacterales* and three (3) *Pseudomonas aeruginosa* strains harboring various classes of carbapenemases (A, B, D) were investigated using both manufacturer’s and modified test protocols to compare results of antibiotic hydrolysis activity and resulting strain classification. Moreover, β-metallo-carbapenemase inhibitors were used to evaluate the usefulness of MBT STAR-Carba test as a susceptibility assay.

## Materials and methods

### Bacterial strains

For this study, 12 *Enterobacterales* and 3 *Pseudomonas aeruginosa* strains (15 in total) belonging to the bacterial collection of the Department of Application Development Microbiology & Diagnostics, Bruker Daltonik GmbH in Bremen (Germany), were used. All strains were previously characterized as β-lactamase-producing bacteria (BLPB) using validated routine PCR methods (TaqMan-probe realtime-PCR using the KAPA™ PROBE FAST qPCR MasterMix Universal (peqlab), Primer and TaqMan-Probes (both metabion) using an ABI 7500 Fast System) and showed the presence of different classes and types of β-lactamases (BLs) coding genes–including Ambler class A & D serine carbapenemases as well as class B β-metallo-carbapenemases (**Table 1**).

**Table 1:** List of investigated carbapenemase-producing bacteria with marked type of β-lactamases and hydrolytic activity value obtained using routine protocol of MBT STAR-Carba assay.

Strain	Type of β-lactamases	Ambler class*	Activity value (According to MBT STAR-Carba Kit protocol)
***P*. *aeruginosa* 1**	GES-5, GES-20	A	0.43
***K*. *oxytoca* 2**	KPC 3	A	1.09
***E*. *coli* 3**	KPC 3	A	1.03
***P*. *aeruginosa* 4**	IMP	B	0.87
***P*. *aeruginosa* 5**	GIM 1	B	0.51
***K*. *pneumoniae* 6**	GIM	B	1.18
***E*. *coli* 7**	NDM-1	B	1.03
***C*. *freundii* 8**	GIM	B	1.00
***C*. *freundii* 9**	VIM	B	0.98
***Salmonella* sp. 10**	IMP-4	B	1.07
***E*. *kobei* 11**	IMP-22	B	0.96
***K*. *pneumoniae* 12**	NDM 1, SHV-28, TEM-1, CTX-M-15, CMY 6, OXA-1, rmtC	B/C	0.94
***K*. *pneumoniae* 13**	OXA-48	D	1.02
***S*. *marcescens* 14**	OXA-1, OXA-181	D	1.03
***E*. *cloacae* 15**	OXA-48	D	0.96
***E*. *coli* ATCC 25922 (Negative control)**			-0.11
***K*. *pneumoniae* ATCC BAA-1705 (Positive control)**			1.06

### Culture conditions

For each analysis, bacterial isolates grown overnight on BD^TM^ Columbia Agar on 5% Sheep Blood plates (Becton Dickinson, Germany) incubated at 35 (±2°C) under aerobic conditions were used. To obtain single bacterial colonies from primary cultures, the quadrant streaking technique was applied.

### MBT STAR-Carba standard assay

**Fig 1:**
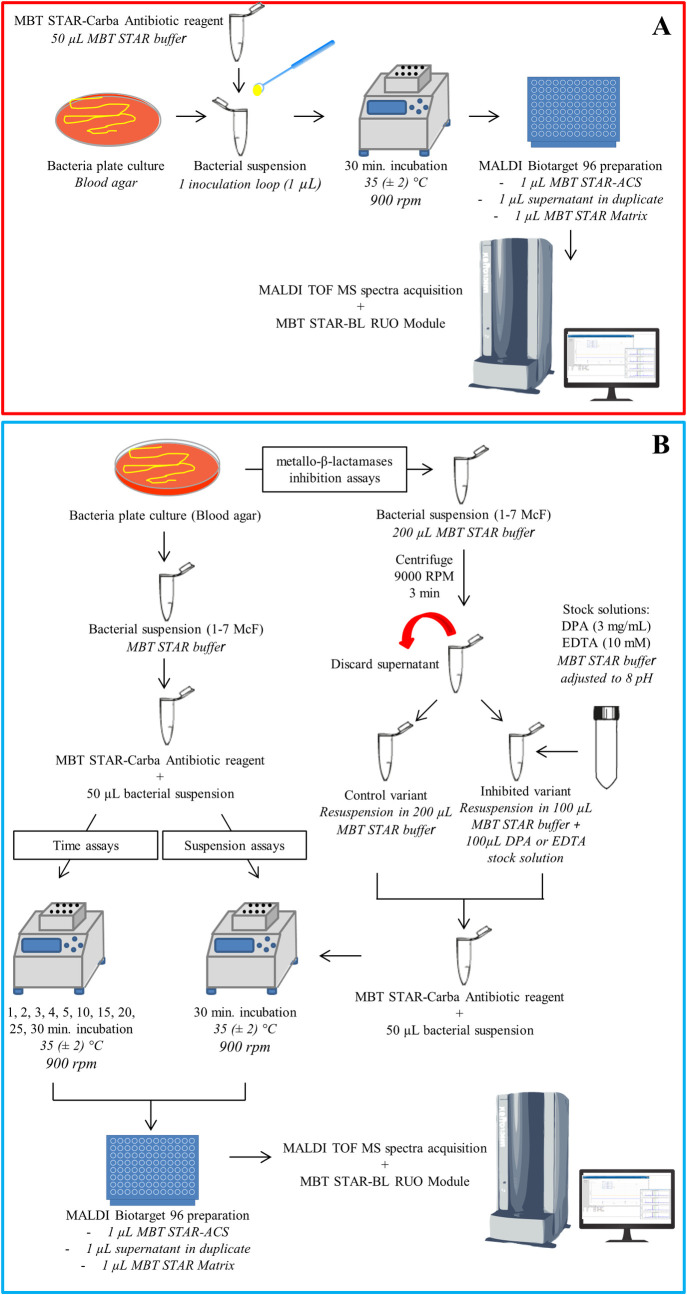
Scheme illustrating the MBT STAR-Carba routine (A) and experimental in-house (B) workflow. DPA—dipicolinic acid; EDTA—ethylenediaminetetraacetic acid.

For testing BLs activity in standard mode, the MBT STAR-Carba assay was performed according to the manufacturer’s instructions provided with MBT STAR-Carba IVD Kit (**Fig 1A**).

For each sample and controls, one inoculation loop (1 μL) filled with sufficient material of bacterial colonies was suspended in the antibiotic solution (MBT STAR-Carba Antibiotic Reagent dissolved in 50 μL MBT STAR Buffer) and then incubated for 30 min. at 35 ± 2°C with shaking (900 rpm) using a Thermomixer (Eppendorf AG, Germany). Subsequently, 1 μL of each sample was deposited on the MALDI Biotarget 96 (Bruker Daltonics) in duplicate, air-dried, and overlaid with 1 μL of MBT STAR Matrix. For each analyzed MALDI Biotarget 96 prepared, one spot with 1 μL MBT STAR-ACS (antibiotic calibration mass standard containing dedicated masses below m/z = 1000) as well as negative (dedicated β-lactam non-hydrolyzing quality control strain—*E*. *coli* ATCC 25922) and positive controls (dedicated β-lactam hydrolyzing quality control strain—*K*. *pneumoniae* ATCC BAA-1705) were used.

### Effect of suspension density and incubation time on the MBT STAR-Carba results–in-house protocols

Multiple bacterial suspensions of different densities (1, 1.5, 2, 2.5, 3, 3.5, 4, 5, 6, 7 McFarland Units—McF) were prepared by transferring and suspending sufficient amounts of respective bacterial colonies from overnight cultures into MBT STAR Buffer. Density was measured by McFarland densitometer DEN-1B (Grant Instruments Ltd, United Kingdom). Subsequently, 50 μL of individual bacterial suspensions were added to the tubes containing dissolved MBT STAR-Carba Antibiotic Reagent and, after mixing, were subjected to (a) 30 min. incubation–suspension assay–and (b) 1, 2, 3, 4, 5, 10, 15, 20, 25, 30 min–incubation time assay–at 35 ± 2°C with shaking (900 rpm) using Thermomixer (Eppendorf AG, Germany) (**Fig 1B**). Afterwards, MALDI Biotarget 96 was prepared according to the procedure described in the previous paragraph.

### β-metallo-carbapenemases inhibition assay

For investigation of β-metallo-carbapenemases inhibition: (a) 3 mg/mL dipicolinic acid (DPA, Sigma Aldrich, Germany) and (b) 10 mM ethylenediaminetetraacetic acid (EDTA, Sigma Aldrich, Germany) stock solutions in MBT STAR Buffer adjusted to 8 pH were used. From overnight cultures of 9 investigated strains characterized by the presence of MBL, bacterial suspensions were prepared as described in the previous paragraph—the McF range used for each strain was selected individually based on previous suspension test results. 200 μL of each suspension were centrifuged (900 rpm, 3 min.), supernatants were discarded and remaining cell pellets were resuspended in (a) 200 μL of MBT STAR Buffer—control variant–and (b) 100 μL of MBT STAR Buffer + 100 μL of respective inhibitor stock solutions: DPA (final conc. 1.5 mg/mL) or EDTA (final conc. 5 mM)—inhibited variants (**Fig 1B**). Subsequently, 50 μL of prepared suspensions were added to the tubes containing dissolved MBT STAR-Carba Antibiotic Reagent, vortexed, incubated (30 min., 35 ± 2°C, 900 rpm) and transferred onto MALDI Biotarget 96 according to procedure described in the paragraph *2*.*3*. Additionally, all tested MBL strains were subjected to control test using non-MBL inhibitor addition—phenyl boronic acid (PBA) at final concentration 2 mg/mL.

### MALDI-TOF MS analysis

Prepared MALDI Biotarget 96 plates were analyzed using a Microflex LT/SH instrument (Bruker Daltonics) and the MBT Compass platform with MBT STAR-BL Module (Bruker Daltonics). Each spot was analyzed in duplicate, finally obtaining 4 spectra for each sample. All the processes, spectra collection, data acquisition, and processing, were performed in a fully automated manner by the software using predefined methods provided by the manufacturer. Obtained results are finally expressed as normalized logRQ values, meaning the calculated logarithm of the ratio of the summed signal intensities of hydrolyzed forms to the summed signal intensities of non-hydrolyzed forms of the antibiotic, normalized using the respective positive (hydrolyzing) and negative (non-hydrolyzing) control strains. The results are understood as a measure of hydrolysis efficiency (higher normalized logRQ = higher degree of antibiotic hydrolysis), with normalized logRQ values ≤ 0.2 threshold—negative result; ≥ 0.4 threshold—positive results; values between these thresholds—unclear results. In this study, all result values described or depicted as “logRQ” refer to normalized values.

### Ranking the strains according to carbapenemases activity

For classification of the tested strains according to antibiotic hydrolyzing activity, principal component analysis (PCA) using the Statistica software package (Statistica ver. 12, Statsoft, Poland) was performed. As variables used: (1) the average values of normalized logRQ obtained for different bacterial suspensions (1–7 McF); (2) the incubation time point in which noted first positive results of hydrolyzation at OD 4 McF (H 4 McF) and at OD 7 McF (H 7 McF) as well as the maximum of hydrolyzation level at OD 4 McF (Hmax 4 McF) and at OD 7 McF (Hmax 7 McF). Before PCA, all investigated data have undergone standardization using Z normalization method in order to avoid outlier issues. Assigning ranks corresponding to the level of carbapenemase activity was performed based on the grouping of the strains on the first two PCs-plane.

## Results

### Results of MBT STAR-Carba standard protocol

**Fig 2:**
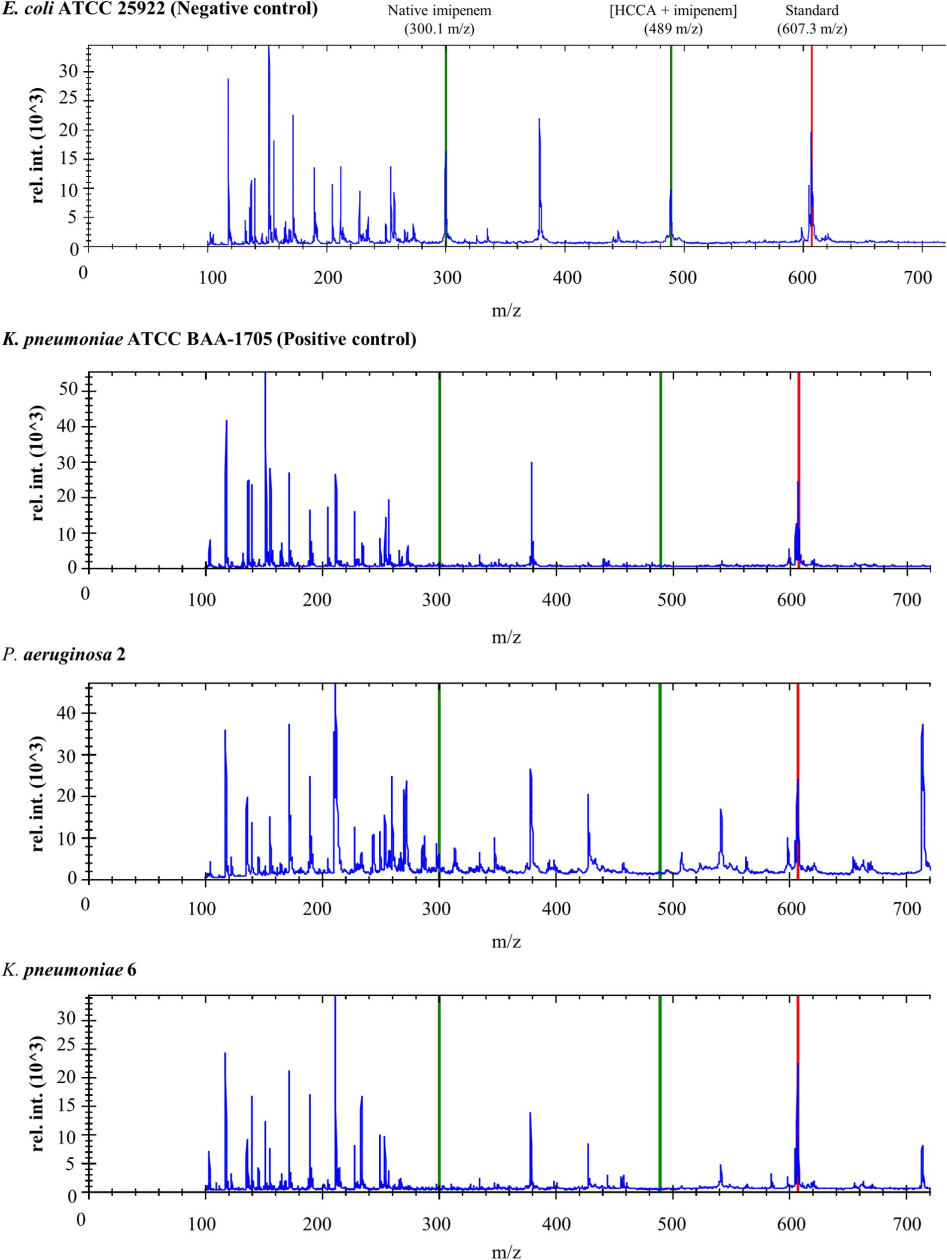
Exemplary MS spectra obtained during detection of carbapenemase activity via design MALDI TOF approach. Green lines represent signals for nonhydrolyzed antibiotic (native and adducts with HCCA) and the red line marks internal standard used for hydrolyzation rate calculation.

The principle of designed approach relies on the mass spectrometric evaluation of the carbapenem molecule after a short co-incubation with the bacterial strains, monitoring the distinct mass peaks of the hydrolyzed and non-hydrolyzed forms of the antibiotic resulting from carbapenemase-dependent β-lactam ring degradation [24,25]. Due to the low signal intensities of the typically observed hydrolyzed molecular forms (254 m/z), the internal standard provided with the MBT STAR Matrix is used as hydrolysis mass reference to calculate the hydrolyzation ratio. On presented spectra (**Fig 2**) it can be observed signals (green lines) representing native imipenem (300.1 m/z) and its adducts with matrix (489 m/z) which intensity vary depend on carbapenemase activity—the lower signal the higher hydrolytic activity.

The design MALDI TOF assay gave correct results for all investigated strains, however, the analysis of results did not reveal a grouping of the investigated strains because of the class of carbapenemases. Mostly, *Enterobacterales* strains representing the same genus had similar activity values–two *Enterobacter* strains, *E*. *kobei* and *E*. *cloacae* (0.96, each), two *C*. *freundii* strains (0.98 and 1.00), and two *E*. *coli* strains (1.03, each). More variance was observed for the *Klebsiella* genus (0.94 to 1.18) (**Table 1**). The use of standard protocol allowed division of hydrolyzing strains into two main groups in view of their carbapenemases activity: (1) strains with considerably lower logRQ values—3 *Pseudomonas aeruginosa* and (2) those demonstrated higher logRQ values–*Enterobacterales* members. Within the first group logRQ values ranged from 0.43–0.87 obtaining the lowest values for GES carbapenemase-producing *P*. *aeruginosa* 1 strain. LogRQ values in the second group ranged from 0.94 (multi-carbapenemase-producers *K*. *pneumoniae* 12) to 1.18 (GIM carbapenemase-producing *K*. *pneumoniae* 6).

### Impact of suspension density on the MBT STAR-Carba results

The tested McF density series of bacterial suspensions ranging from McF 1 to 7 enabled the indication of a present carbapenemase activity in all respective strains except for GES producing *P*. *aeruginosa* 1, for which an increase in logRQ values with increasing suspension density has been observed; however, none exceeded the 0.2 threshold (**Table 2; S1 Data**).

**Table 2:** The hydrolytic activity levels (normalized logRQ) noted for the investigated bacteria depending on the cell’s suspension densities (1–7 McF).

		Hydrolytic activity level [normalized logRQ]										
	Strain McF	1.0	1.5	2.0	2.5	3.0	3.5	4.0	5.0	6.0	7.0	Δcontrols
	***P*. *aeruginosa* 1**	**-0.40**	**-0.40**	**-0.43**	**-0.41**	**-0.40**	**-0.37**	**-0.28**	**-0.27**	**-0.06**	**-0.06**	**1.12**
**A**	***K*. *oxytoca* 2**	**0.08**	**0.10**	**0.13**	**0.18**	**0.12**	**0.30**	**0.39**	**1.02**	**1.05**	**0.99**	**1.90**
	***E*. *coli* 3**	**0.24**	**0.73**	**0.85**	**0.86**	**0.90**	**0.94**	**0.90**	**0.97**	**1.00**	**1.00**	**1.67**
	***P*. *aeruginosa* 4**	**-0.36**	**-0.34**	**-0.36**	**-0.29**	**0.44**	**0.74**	**0.98**	**0.73**	**0.58**	**0.55**	**1.12**
	***P*. *aeruginosa* 5**	**0.10**	**0.74**	**1.00**	**1.02**	**0.97**	**0.92**	**0.80**	**0.63**	**0.48**	**0.37**	**1.40**
	***K*. *pneumoniae* 6**	**0.83**	**0.80**	**0.82**	**0.91**	**0.97**	**0.91**	**0.97**	**1.00**	**1.08**	**1.06**	**1.71**
	***E*. *coli* 7**	**0.80**	**0.87**	**0.88**	**0.95**	**0.83**	**0.92**	**0.89**	**0.93**	**0.96**	**0.97**	**1.90**
**B**	***C*. *freundii* 8**	**0.02**	**0.46**	**0.55**	**0.60**	**0.86**	**0.93**	**0.96**	**0.96**	**0.98**	**1.09**	**1.67**
	***C*. *freundii* 9**	**0.10**	**0.07**	**0.05**	**0.06**	**0.13**	**0.15**	**0.26**	**1.02**	**1.04**	**1.02**	**1.81**
	***Salmonella* sp. 10**	**0.16**	**0.86**	**0.90**	**0.91**	**0.93**	**0.84**	**0.99**	**1.04**	**1.06**	**1.03**	**1.77**
	***E*. *kobei* 11**	**0.82**	**0.85**	**0.86**	**0.90**	**0.95**	**0.90**	**0.92**	**1.00**	**1.03**	**1.04**	**1.71**
	***K*. *pneumoniae* 12**	**0.88**	**0.91**	**0.96**	**0.95**	**0.90**	**0.98**	**1.03**	**1.12**	**1.04**	**0.98**	**1.77**
*** *D**	***K*. *pneumoniae* 13**	**0.06**	**0.05**	**0.06**	**0.11**	**0.03**	**0.99**	**1.05**	**1.05**	**1.12**	**1.10**	**1.60**
	***S*. *marcescens* 14**	**0.01**	**0.06**	**0.00**	**0.06**	**0.13**	**0.33**	**1.04**	**1.03**	**1.03**	**1.02**	**1.67**
	***E*. *cloacae* 15**	**0.12**	**0.08**	**0.21**	**0.32**	**0.68**	**0.99**	**1.03**	**1.02**	**1.03**	**1.04**	**1.81**
**non-hydrolyzed**												
**unclear results**												
**hydrolyzed**		**0.41–0.65**		**0.66–0.89**		**>0.90**						

Δ controls—quality control of the results calculated by subtracting the highest calculated original logRQ value of the acquired negative control spectra from the lowest calculated original logRQ value of the acquired positive control spectra; ≥ 0.7 means that the assay results are evaluated as accepted.

**Fig 3:**
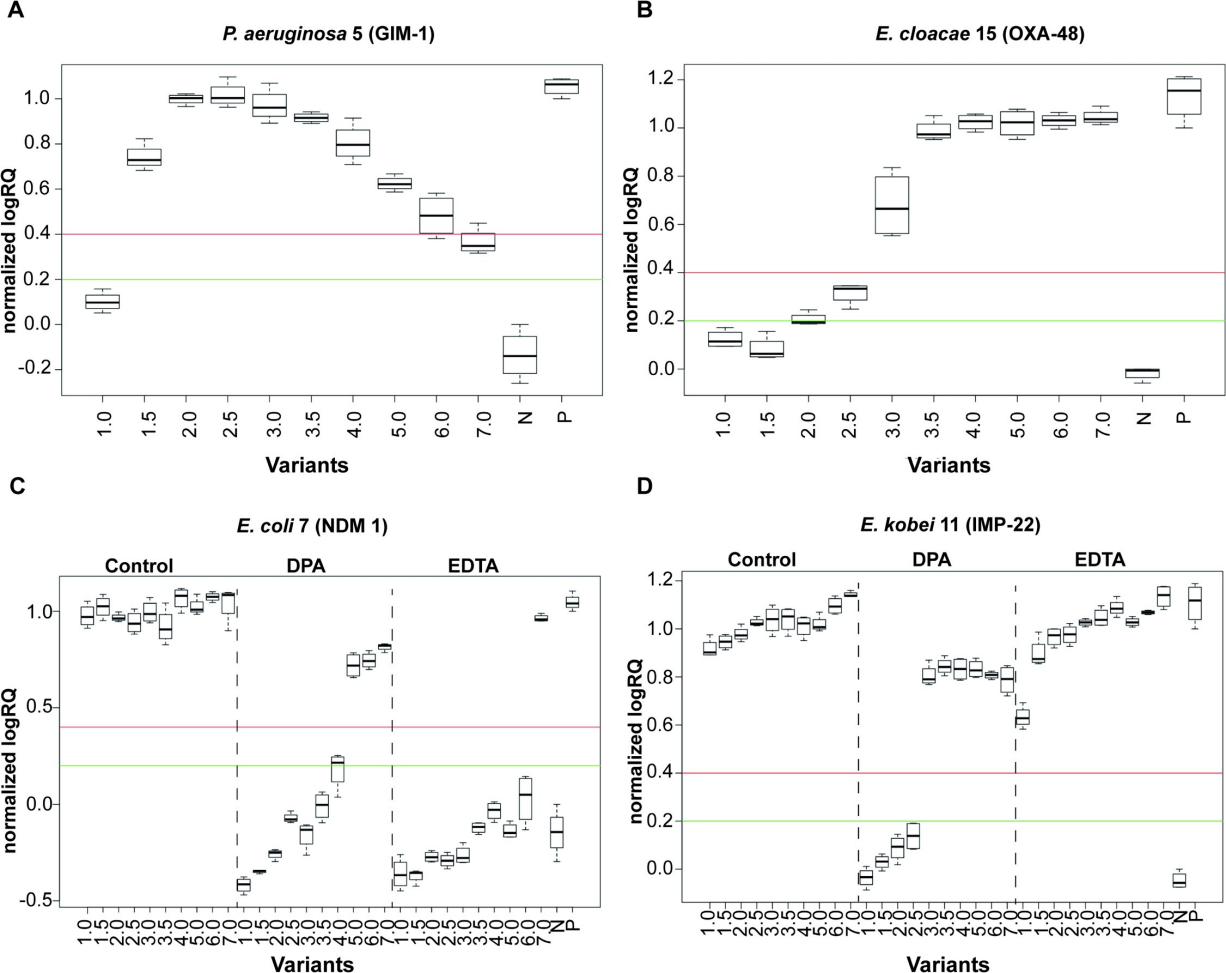
Examples of graphs showing the dependence of the determined carbapenemase activity level (normalized logRQ) on the density of the cell suspension used (1–7 McF) (A, B) as well as the addition of the inhibitor (DPA and EDTA) (C, D) created using MBT Biotyper Prototype software. N- negative control; P–positive control. The graph area between the green and red lines (0.2–0.4 normalized logRQ values) determines the range of the unclear results of hydrolytic activity.

Examples of graphs obtained using MBT Biotyper Prototype software during results generating are presented in **Fig 3A and 3B**. The analysis of the results revealed a different effect of bacterial density on the logRQ values depending on the bacterial order and class of carbapenemase.

**Fig 4:**
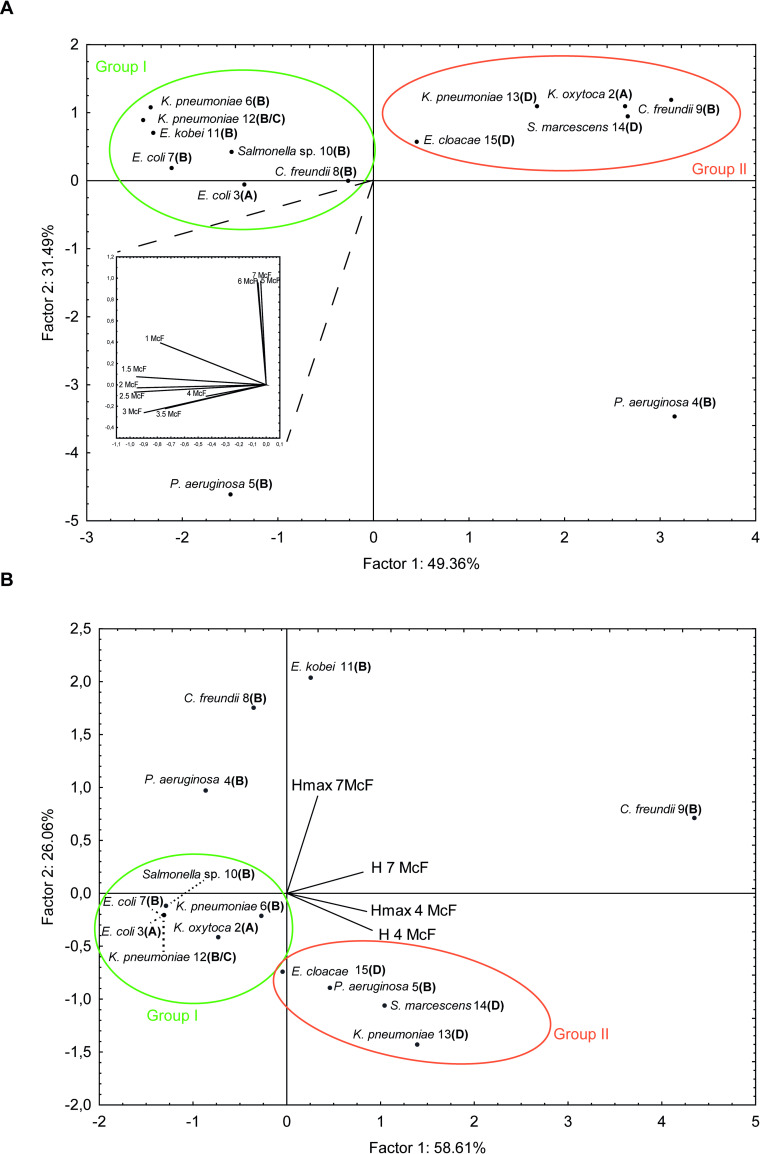
Grouping of the bacterial strains on the factor-plane in view of logRQ values at different suspensions (A) and incubation time needed to observe positive and maximal results of hydrolytic activity (B).

Regarding *Enterobacterales*, division of the strains into two groups could be observed (**Fig 4A**).

Group I was mostly comprised of β-metallo-carbapenemase-producing strains (6 out of 7) demonstrating hydrolytic activity in the whole McF range and recognized as the most active group among investigated bacteria. In 4 cases–*E*. *coli* 7, *K*. *pneumoniae* 6/12 as well as *E*. *kobei* 11—positive detection of carbapenemases activity was possible already with the lowest suspension density (1 McF) and differences between 1 and 7 McF suspensions did not exceed 0.25 logRQ. Group II was mostly represented by class A and D carbapenemase producers (4 out of 5 strains) characterized by high hydrolytic activity only at a higher McF range–positive results of the test started from 3–5 McF depending on the strain (**Table 2**).

For all investigated *Enterobacterales* strains, elevated logRQ values were observed as suspension density increased. In turn, for two β-metallo-carbapenemase-producing *P*. *aeruginosa* strains (4 and 5), a considerably different behavior was observed. For both strains, the increase of hydrolytic activity in the lower suspension density range was followed by a gradual activity decrease for respective higher McF values. For *P*. *aeruginosa* 4, positive results started with a 3 McF suspension achieving the highest logRQ value at 4 McF before decreasing again, while *P*. *aeruginosa* 5 strain demonstrated first positive carbapenemase activity at 1.5 McF and maximum values in the range of 2–3 McF.

Regardless of the family investigated, as the bacterial suspension density increased, the logRQ values of the tested strains approached the levels obtained when using the standard protocol, except for the strain *P*. *aeruginosa* 4 in which standard method normalized logRQ values placed between results noted for 4 and 5 McF suspension (**Table 2**).

### Effect of incubation time on the MBT STAR-Carba results

Based on the suspension experiments, bacterial suspensions with McF 4 and 7 were chosen for investigation of the incubation time. Strain *P*. *aeruginosa* 1 was excluded from investigation due to lack of positive results in investigated suspensions range.

Regarding lower suspension density, the analysis revealed that in case of all OXA-like carbapenemase producers (class D) as well as *P*. *aeruginosa* 3 strain time needed to achieve positive results (H 4McF) was longer compared to the class A and B producers except for *C*. *freundii* 9 (no positive results at 4 McF noted) (Group II, **Fig 4B; S2 Data**). The same applies to time to obtain maximum hydrolyzation level (H_max_ 4 McF). Strains clustered as Group I on the PCA graph were characterized by the shortest time needed to achieve positive as well as maximum results of hydrolytic activity and was represented by all class A producers as well as almost half of the β-metallo-carbapenemases producing strains (4 out of 9). Analysis revealed that those strains required no more than 3 minutes of incubation time to give positive results when OD 4 McF was used while in the case of class D producers 10 and more minutes were needed (**S1 Table; S2 Data**).

Considering the highest bacterial density (7 McF), the analysis did not reveal differences between the class of produced carbapenemases since in all cases except for *C*. *freundii* 9 (4 min) the first time point which revealed positive results was 1 min (**S1 Table**). Instead, differences were noted in case of time needed to achieve maximum hydrolyzation level, where, 4 of class B producing strains—*P*. *aeruginosa* 4, *C*. *freundii* 8/9, and *E*. *kobei* 11, reached the highest logRQ after at least 15 min. of incubation. In the case of the rest strains H_mas_ 7 McF did not exceeded 5 minutes.

### Ranking the strains

Based on the PCA results, strain ranking related to the levels of hydrolytic activity in comparison with results of the standard procedure was performed (**Table 3**).

**Table 3:** Comparison of strain ranking based on the results obtained during suspension assay and time assay as well as standard protocol. Compliance refers to the similarities with standard method.

	Ranking			
Strain	Suspension	Time	Total	Standard
***P*. *aeruginosa* 1(A)**	15	na	**15**	**15**
***K*. *oxytoca* 2(A)**	10	5	**7**	**2**
***E*. *coli* 3(A)**	6	3	**4**	**4**
***P*. *aeruginosa* 4(B)**	14	11	**13**	**13**
***P*. *aeruginosa* 5(B)**	13	8	**11**	**14**
***K*. *pneumoniae* 6(B)**	1	7	**3**	**1**
***E*. *coli* 7(B)**	4	2	**2**	**4**
***C*. *freundii* 8(B)**	7	12	**10**	**8**
***C*. *freundii* 9(B)**	12	14	**14**	**9**
***Salmonella* sp. 10(B)**	5	4	**4**	**3**
***E*. *kobei* 11(B)**	3	13	**8**	**10**
***K*. *pneumoniae* 12(B/C)**	2	1	**1**	**12**
***K*. *pneumoniae* 13(D)**	9	9	**9**	**4**
***S*. *marcescens* 14(D)**	11	10	**11**	**4**
***E*. *cloacae* 15(D)**	8	6	**6**	**10**
**% Good compliance (Δ 0–2)**	60	33	**53**	
**% Weak compliance (Δ ≥ 3)**	40	67	**47**	

Based on the total ranking, the analysis showed good compliance between tested and standard protocol in 53% of cases and decreased to 33% if only time ranking is accounted for. Ranking based on the suspension assay was similar to standard ranking; however, for 40% of the strains only weak compliance was revealed. The best rank matching was obtained for *Pseudomonas aeruginosa*, *E*. *coli*, and *Salmonella* sp. 10 strains while the highest discrepancies were detected in the case of multi-carbapenemase-producer *K*. *pneumoniae* 12 ranked as 1 and 12 the most active strain according to the experimental test and standard procedure, respectively.

### Results of β-metallo-carbapenemases inhibition assay

The use of the MBT STAR-Carba test to track the effect of carbapenemase inhibition in the tested range of cell suspension density revealed great differences between the type of inhibitor used as well as type of β-metallo-carbapenemases (**Table 4**). Exemplary graphs created via MBT Biotyper Prototype software are presented in **Fig 3C and 3D**. Moreover, a control test with the use of a non-MBL inhibitor– 2 mg/mL PBA–did not reveal any significant inhibitory effects (**S2 Table**), thereby confirming that the noted observation actually reflects a DPA and EDTA influence and that they are not an artifact.

**Table 4:** Decrease in the level of hydrolytic activity of the metallo-β-lactamases producing strains under DPA and EDTA addition depending on the bacterial suspension density.

	Significant inhibition									
	**Under DPA addition**									
**Strain McF**	**1.0**	**1.5**	**2.0**	**2.5**	**3.0**	**3.5**	**4.0**	**5.0**	**6.0**	**7.0**
*P*. *aeruginsa* 4 (IMP)										
*Salmonella* sp. 10 (IMP-4)										
*E*. *kobei* 11 (IMP-22)										
*P*. *aeruginsa* 5 (GIM-1)										
*K*. *pneumoniae* 6 (GIM)										
*C*. *freundii* 8 (GIM)										
*E*. *coli* 7 (NDM-1)										
*K*. *pneumoniae* 12 (NDM-1)										
*C*. *freundii* 9 (VIM)										
	**Under EDTA addition**									
	**1.0**	**1.5**	**2.0**	**2.5**	**3.0**	**3.5**	**4.0**	**5.0**	**6.0**	**7.0**
*P*. *aeruginsa* 4 (IMP)										
*Salmonella* sp. 10 (IMP-4)										
*E*. *kobei* 11 (IMP-22)										
*P*. *aeruginsa* 5 (GIM-1)										
*K*. *pneumoniae* 6 (GIM)										
*C*. *freundii* 8 (GIM)										
*E*. *coli* 7 (NDM-1)										
*K*. *pneumoniae* 12 (NDM-1)										
*C*. *freundii* 9 (VIM)										

Black–excluded from the calculation due to negative results in control; blue- significant decrease compared to the control variant—normalized logRQ values <0.2 (no hydrolysis); yellow—significant decrease compared to the control variant—normalized logRQ values 0.2–0.4 (unclear results); orange—significant decrease compared to the control variant—normalized logRQ values >0.4 (hydrolysis); white–insignificant differences. DPA—dipicolinic acid; EDTA—ethylenediaminetetraacetic acid.

Considering inhibitors, tested strains proved to be typically more sensitive to 1.5 mg/ml DPA addition–with a significant decrease in logRQ values in all strains regardless of the suspension density used (**Table 4; S3 Data**). Decline in the hydrolytic activity within the investigated McF range differed depending on the produced type of carbapenemase. GIM producers appeared to be the most sensitive to DPA presence since in almost all cases caused the test result to change from positive to negative except for 6 and 7 McF suspensions of *K*. *pneumoniae* 6 where unclear results were noted. Simultaneously, the same strains were less sensitive to the addition of EDTA, showing a significant decrease in the logRQ values only in 3 cases, including one negative result. EDTA demonstrated higher inhibitory effect than DPA only in the case of one strain–NDM-1 producing *E*. *coli* 7 –negative results up to 6 McF compared to 3.5 McF that of DPA.

Regarding individual strains, *E*. *kobei* 11 was the least sensitive to the presence of inhibitors—a decrease in logRQ below 0.2 threshold (which means no hydrolysis) only under supplementation with DPA in the lower McF range (up to 2.5 McF). The greatest inhibitory effect was observed for NDM-like carbapenemase producing *E*. *coli* 7 and *K*. *pneumoniae* 12 as well as in case of *C*. *freundii* 9; however, the latter one demonstrated relatively low overall hydrolytic activity which was starting only from a 5 McF suspension.

## Discussion

Design spectrometric approach proved to be a universal, rapid, and highly sensitive tool for detecting present hydrolytic activity amongst the range of carbapenemases tested, including class A, B, and D. Also, class B GES type carbapenemases were detected, albeit well known to exhibit relatively low hydrolytic activity and therefore to challenge many current colorimetric tests (e.g. β-Carba Test, Biorad) [26,27]. Although the results obtained by the standard procedure revealed great differences in activity levels between individual *Enterobacterales* and *Pseudomonas* strains–with typically *P*. *aeruginosa* strains being characterized by lower logRQ values–the test does not allow to ascribe differences in activity to different types or classes of carbapenemases. In this study, carbapenemase-type specific hydrolytic differences were revealed by modifying the standard test procedure regarding the amount of bacterial material, incubation time, and application of inhibitors. In most cases, there is a clear relationship of the modified parameters and the detected activity with the standard procedure being typically in an optimal range for sensitive detection. In most cases, logRQ values of 7 McF variant closed to that obtained using 1 uL inoculation loop which suggests transferring the similar number of cells. Indeed, 7 McF suspension density corresponds to ca. 3 x 10^8^ colony forming units (cfu) and theoretical concentration in 1 uL inoculation loop ranged 3–6 x 10^8^ CFU/mL as followed manufacturer guidelines. For GES-harbouring *P*. *aeruginosa* strain, only the standard procedure was detecting the carbapenemase and false-negative results were noted when the modified procedure was applied what resulted from the lower amount of bacterial cells and indicated the reduced activity of this type of carbapenemases. Comparing other types, most metallo-type class B carbapenemase-producing bacteria demonstrated relevant hydrolytic activity also in a wider McF range compared to the class D producers tested, a class of β-lactamases also known to be difficult for rapid detection [28]. Moreover, strains for which positive results were observed already at the lowest density tested (1 McF) and thus classified as the most active, also belonged to the class B producers. Burckhardt et al. [29] noted that strains producing carbapenem-hydrolyzing class D (OXA-type) beta-lactamases demonstrate weaker imipenem hydrolytic activity compared to the MBL producers. Our findings are in agreement with the general statement that strains producing class D β-lactamases, especially OXA-48 producers, are considered as difficult-to-detect strains since they are characterized by low carbapenem MICs and often go undetected by most phenotypic methods [30,31]. Nevertheless, the use of design spectrometric assay can effectively help not only to reliably detect the activity of oxacillinases, but also in their further classification. Similar effect was achieved by Álvarez-Buylla et al. [32] using dipicolinic acid and Zn^2+^ supplementation. Interestingly, similar classification of the strains into the metallo- and OXA-like producers can be performed based on the time needed to present relevant hydrolytic activity, where the oxacillinases-producing strains happened to need longer time-to-result (**Fig 4B**).

The observed lower hydrolytic activity of *P*. *aeruginosa* strains compared to that in *Enterobacterales* has previously been reported in the literature and was associated, among others, with lower quality of carbapenemase producing *P*. *aeruginosa* spectra [20]. Results of our studies suggest that the explanation of such findings could also be a different behavior of *P*. *aeruginosa* strains under increasing suspension density compared to *Enterobacterales* members. Possible explanation of such phenomenon could be the fact that *P*. *aeruginosa* has the genetic capacity to express a wide repertoire of carbapenems resistance mechanisms—besides enzymatic antibiotic degradation also expression of efflux pumps and low outer membrane permeability [33,34]. Moreover, Potron et al. [35] claim that carbapenem resistance in *P*. *aeruginosa* is mostly related to porin deficiency. In this context, the observed gradual decrease of hydrolytic activity with increasing suspension density in higher OD range may be due to the superiority of the efflux resistance mechanism and lowering membrane permeability leading to the limitation of the intracellular penetration of carbapenems. A similar observation was noted in the work of Miltgen et al. [36], in which disruption of the outer membrane by high concentrations of colistin and a freezing-thawing step was proposed as a solution for improving detection performance. *P*. *aeruginosa* is also known for their ability to synthesize significant amounts of siderophores demonstrating an affinity for Zn^2+^ [37] which could affect the availability of this metal for carbapenemases. However, the available literature lacks any work that addresses the impact of siderophore synthesis on carbapenemases activity detection. Due to restricted number of investigated *P*. *aeruginosa* strains drawing of general findings is limited, nevertheless, our findings prove a great need to deepen studies on the carbapenemases activity within *Pseudomonas* species conducted on the bigger scale and applying various conditions like different amount of both Zn^2+^ and biomass.

Multiple studies evaluated the time needed for reliable carbapenemases activity detection via MALDI-TOF MS [29,32,38]. However, all of them involve homebrew, non-standardized protocols. Our studies revealed that for the vast majority of bacteria tested (13 out of 15) incubation in the presence of the benchmark antibiotic could be shortened to 1 min if McF 7 suspension is used, a value also reached by the standard procedure. This applies to both *Enterobacterales* and *P*. *aeruginosa* strain. Considering that in almost half of the investigated cases, 1 min of incubation was enough to reveal the maximum logRQ value, we can suppose that enzymatic degradation of carbapenem molecules occurred immediately after mixing bacterial suspension with antibiotic solution. Therefore, the spectrometric assay could be potentially used for reliable carbapenemases detection in many cases of *Enterobacterales* and *Pseudomonas* strains, almost omitting the incubation step making this test even shorter. Similarly, Chong et colleagues [39] revealed complete imipenem hydrolization right after reaction mixture preparation (T = 0) for 85% of class A carbapenemase-producers, however, the majority of class B and D carbapenemase-producers did not fully hydrolyze imipenem until the 2 h of incubation. Such discrepancies may result from different reaction buffer composition and the applied mass spectrometric detection compared to that in our studies. As Knox and Palombo [30] observed, the addition of zinc ions is essential for consistent detection of many class B carbapenemases producers, like NDM- and VIM-harbouring strains and generally should be a requirement during the investigation of metallo-carbapenemase producers in these assays.

This approach has become particularly important in regard of increasing use of MALDI-TOF MS in clinical microbiology. Results of the works from the last few years regarding the use of MALDI TOF MS analysis for investigation of different carbapenemase activities—under addition of MBL inhibitors (e.g. ertapenem hydrolysis by IMP- and VIM-harbouring enterobacteria and *P*. *aeruginosa* using 50 mg/mL EDTA [40], by NDM-producing *K*. *pneumoniae* using 0.05 mg/mL DPA [3] or by VIM- and NDM-like *K*. *pneumoniae* as well as VIM-like *P*. *aeruginosa* using 1.5 mg/mL DPA [41]) makes this methodology a good option as a tool for the evaluation of new effective combinations of β-lactams and their inhibitors in any modern laboratory routine. Nevertheless, the authors referred to the use of ertapenem, which implicates the need to conduct a relatively long incubation to obtain correct results—up to 2 hours. Similarly to our work, recent studies of Oviano et colleagues [42,43] showed that the use of imipenem gives the opportunity to obtain reliable results of susceptibility testing in a simple way already after 30 minutes as it was in the case of combinations: imipenem–avibactam and imipenem-relebactam. Although results of mentioned works showed high sensitivity (up to 98%) and specificity (up to 100%) in detecting KPC- producing *Enterobacterales* susceptible to imipenem/relebactam [43] as well as for detecting KPC and OXA-48-type isolates through avibactam inhibition [42], authors faced the obstacles with the repeated indeterminate results for certain strains or inability to distinguish between the types of carbapenemases. As we suppose, those limitations could be associated with the use of 1 uL microbial loop containing only an approximate number of cells. Contrary to this, in our studies, we used the range of bacterial suspension densities which enabled revealing differences in susceptibility between types of β-metallo-carbapenemases and inhibitors. Thus, conducting tests in a wide range of certain suspension densities opens the possibility of sensitivity comparisons between strains on a given inhibitor depending on the type of carbapenemase produced, solve the problem of indeterminate results as well as has the potential to facilitate interlaboratory comparisons. Considering the fact that special emphasis is currently placed on discovering new clinically significant MBL inhibitors like VNRX-5133 (novel boronic acid, phase I), ANT431 (novel pyridine-2-carboxylic acid, preclinical), or potentially also avibactam (phase III) the development of which is at different phases of clinical trials [44], our proposed use of design assay demonstrates high potential as a surrogate test for susceptibility before the microdilution results become publicly available. In the future investigation, inhibition effect of the other classes of carbapenemases (A, D) using the spectrometric assay is planned, since our findings suggest, as it was in the case of MBL, it may turn out to be a reliable and fast tool for clinical diagnostic laboratories also in this field.

## Conclusions

The new spectrometric assay has become known as a highly sensitive and robust tool for a very rapid detection of carbapenemases, including the broad spectrum of β-metallo-carbapenemases. The outcome of our research indicates that, besides using the standard workflow, providing highly sensitive results, procedural modifications of this assay, like varying suspension densities and incubation times, demonstrate great potential in further sub-characterization of samples. The investigation of activity level differences based on normalized logRQ values allowed not only the differentiation of various bacterial species on the order level but also a correlation of different classes of carbapenemases, even within the class B metallo β-lactamases. It also proved that the application of a defined suspension density gives the opportunity to shorten incubation time and thus the total time-to-result of analysis along with further increasing knowledge about differences in the antibiotic hydrolysis level and sensitivity to inhibitors addition of an investigated strain. Such information could be useful during initial adaption towards a proper antibiotic therapy as well as during development of new MBL inhibitors.

## Supporting information

S1 Table The shortest incubation time needed to observe hydrolytic activity using MBT STAR-Carba assay depending on the bacterial suspension density (4 and 7 McF). H–first time point with pronounced hydrolysis (positive results); Hmax- time point with maximum hydrolysis achieved.
(DOCX)

S2 Table Result of control test of effect of non-MBL inhibitor (phenyl boronic acid -PBA) addition on the level of hydrolytic activity of the investigated strains representing the Ambler class B metallo-β-lactamases depending on the bacterial suspension density. ΔlogRQ—differences between the normalized logRQ of the control variants and treated ones. Negative values mean increase compare to control. In case of results recognized as”non-hydrolyzed” in control variants–values are not shown.
(DOCX)

S1 Data The results of the hydrolytic activity measurements depending on the cells suspension densities". nhydrInt 300.2"—the signal intensity of the unhydrolyzed imipenem [M+H]+; "nhydrInt 489"—the signal intensity of undhydrolyzed imipenem in an adduct form; "calInt"—intensity of the calibrator used (m/z 607.3); "deltaC"—differences between logRQ of positive and negative control.
(CSV)

S2 Data The result of hydrolytic activity measurements under different incubation time [1–30 minutes]". nhydrInt 300.2"—the signal intensity of the unhydrolyzed imipenem [M+H]+; "nhydrInt 489"—the signal intensity of undhydrolyzed imipenem in an adduct form; "calInt"—intensity of the calibrator used (m/z 607.3); "deltaC"—differences between logRQ of positive and negative control.
(CSV)

S3 Data The result of hydrolytic activity measurements for metallo-carbapenemases producing strains under dipiculonic acid [DPA] and [EDTA] addition depending on the bacterial suspension density". nhydrInt 300.2"—the signal intensity of the unhydrolyzed imipenem [M+H]+; "nhydrInt 489"—the signal intensity of undhydrolyzed imipenem in an adduct form; "calInt"—intensity of the calibrator used (m/z 607.3); "deltaC"—differences between logRQ of positive and negative control.
(CSV)

## References

[1] Nordmann P, Poirel L, Dortet L. Rapid detection of carbapenemase-producing enterobacteriaceae. Emerg Infect Dis. 2012;18: 1503–1507. 10.3201/eid1809.120355 22932472 PMC3437707

[2] Bush K. Carbapenemases: Partners in crime. Journal of Global Antimicrobial Resistance. Elsevier Ltd; 2013. pp. 7–16. 10.1016/j.jgar.2013.01.005 27873609

[3] Monteferrante CG, Sultan S, ten Kate MT, Dekker LJM, Sparbier K, Peer M, et al. Evaluation of different pretreatment protocols to detect accurately clinical carbapenemase-producing Enterobacteriaceae by MALDI-TOF. J Antimicrob Chemother. 2016;71: 2856–2867. 10.1093/jac/dkw208 27287232

[4] Albiger B, Glasner C, Struelens MJ, Grundmann H, Monnet DL, Koraqi A, et al. Carbapenemase-producing Enterobacteriaceae in Europe: Assessment by national experts from 38 countries, May 2015. Eurosurveillance. 2015;20. 10.2807/1560-7917.ES.2015.20.45.30062 26675038

[5] Anantharajah A, Tossens B, Olive N, Kabamba-Mukadi B, Rodriguez-Villalobos H, Verroken A. Performance Evaluation of the MBT STAR®-Carba IVD Assay for the Detection of Carbapenemases With MALDI-TOF MS. Front Microbiol. 2019;10. 10.3389/fmicb.2019.01413 31281303 PMC6596351

[6] Gazin M, Paasch F, Goossens H, Malhotra-Kumar S. Current trends in culture-based and molecular detection of extended-spectrum-β-lactamase-harboring and carbapenem-resistant Enterobacteriaceae. Journal of Clinical Microbiology. 2012. pp. 1140–1146. 10.1128/JCM.06852-11 22259207 PMC3318521

[7] Cantón R, Akóva M, Carmeli Y, Giske CG, Glupczynski Y, Gniadkowski M, et al. Rapid evolution and spread of carbapenemases among Enterobacteriaceae in Europe. Clinical Microbiology and Infection. Blackwell Publishing Ltd; 2012. pp. 413–431. 10.1111/j.1469-0691.2012.03821.x 22507109

[8] Choquet M, Guiheneuf R, Castelain S, Cattoir V, Auzou M, Pluquet E, et al. Comparison of MALDI-ToF MS with the Rapidec Carba NP test for the detection of carbapenemase-producing Enterobacteriaceae. Eur J Clin Microbiol Infect Dis. 2018;37: 149–155. 10.1007/s10096-017-3115-4 28980084

[9] Cordovana M, Abdalla M, Ambretti S. Evaluation of the MBT STAR-Carba Assay for the Detection of Carbapenemase Production in Enterobacteriaceae and Hafniaceae with a Large Collection of Routine Isolates from Plate Cultures and Patient-Derived Positive Blood Cultures. Microb Drug Resist. 2020;26: 1298–1306. 10.1089/mdr.2019.0466 32412820

[10] Dortet L, Tandé D, de Briel D, Bernabeu S, Lasserre C, Gregorowicz G, et al. MALDI-TOF for the rapid detection of carbapenemase-producing Enterobacteriaceae: comparison of the commercialized MBT STAR®-Carba IVD Kit with two in-house MALDI-TOF techniques and the RAPIDEC® CARBA NP. J Antimicrob Chemother. 2018;73: 2352–2359. 10.1093/jac/dky209 29897463

[11] Girlich D, Halimi D, Zambardi G, Nordmann P. Evaluation of Etest® strips for detection of KPC and metallo-carbapenemases in Enterobacteriaceae. Diagn Microbiol Infect Dis. 2013;77: 200–201. 10.1016/j.diagmicrobio.2013.08.002 24041554

[12] Pierce VM, Simner PJ, Lonsway DR, Roe-Carpenter DE, Johnson JK, Brasso WB, et al. Modified carbapenem inactivation method for phenotypic detection of carbapenemase production among enterobacteriaceae. J Clin Microbiol. 2017;55: 2321–2333. 10.1128/JCM.00193-17 28381609 PMC5527410

[13] Zhou M, Wang D, Kudinha T, Yang Q, Yu S, Xu YC. Comparative evaluation of four phenotypic methods for detection of class A and B carbapenemase-producing enterobacteriaceae in China. J Clin Microbiol. 2018;56. 10.1128/JCM.00395-18 29769274 PMC6062786

[14] Nordmann P, Naas T, Poirel L. Global spread of carbapenemase producing Enterobacteriaceae. Emerg Infect Dis. 2011;17: 1791–1798. 10.3201/eid1710.110655 22000347 PMC3310682

[15] Tamma PD, Simner PJ. Phenotypic detection of carbapenemase-producing organisms from clinical isolates. Journal of Clinical Microbiology. American Society for Microbiology; 2018. 10.1128/JCM.01140-18 30158194 PMC6204673

[16] Findlay J, Hopkins KL, Meunier D, Woodford N. Evaluation of three commercial assays for rapid detection of genes encoding clinically relevant carbapenemases in cultured bacteria. J Antimicrob Chemother. 2014;70: 1338–1342. 10.1093/jac/dku571 25630646

[17] Oviaño M, Gómara M, Barba MJ, Revillo MJ, Barbeyto LP, Bou G. Towards the early detection of β-lactamase-producing Enterobacteriaceae by MALDI-TOF MS analysis. J Antimicrob Chemother. 2017;72: 2259–2262. 10.1093/jac/dkx127 28444315

[18] Rodríguez-Sánchez B, Cercenado E, Coste AT, Greub G. Review of the impact of MALDI-TOF MS in public health and hospital hygiene, 2018. Eurosurveillance. 2019;24. 10.2807/1560-7917.ES.2019.24.4.1800193 30696525 PMC6351997

[19] Sanguinetti M, Posteraro B. Mass spectrometry applications in microbiology beyond microbe identification: progress and potential. Expert Review of Proteomics. Taylor and Francis Ltd; 2016. pp. 965–977. 10.1080/14789450.2016.1231578 27598407

[20] Hrabák J, Walková R, Studentová V, Chudácková E, Bergerová T. Carbapenemase activity detection by matrix-assisted laser desorption ionization-time of flight mass spectrometry. J Clin Microbiol. 2011;49: 3222–7. 10.1128/JCM.00984-11 21775535 PMC3165603

[21] Sparbier K, Schubert S, Weller U, Boogen C, Kostrzewa M. Matrix-assisted laser desorption ionization-time of flight mass spectrometry-based functional assay for rapid detection of resistance against β-lactam antibiotics. J Clin Microbiol. 2012;50: 927–937. 10.1128/JCM.05737-11 22205812 PMC3295133

[22] Lasserre C, De Saint Martin L, Cuzon G, Bogaerts P, Lamar E, Glupczynski Y, et al. Efficient Detection of Carbapenemase Activity in Enterobacteriaceae by Matrix-Assisted Laser Desorption Ionization-Time of Flight Mass Spectrometry in Less Than 30 Minutes. J Clin Microbiol. 2015;53: 2163–71. 10.1128/JCM.03467-14 25926485 PMC4473196

[23] Ghebremedhin B, Halstenbach A, Smiljanic M, Kaase M, Ahmad-Nejad P. MALDI-TOF MS based carbapenemase detection from culture isolates and from positive blood culture vials. Ann Clin Microbiol Antimicrob. 2016;15. 10.1186/s12941-016-0120-x 26839024 PMC4736273

[24] Cordovana M, Kostrzewa M, Glandorf J, Bienia M, Ambretti S, Pranada AB. A full MALDI-based approach to detect plasmid-encoded KPC-producing klebsiella pneumoniae. Front Microbiol. 2018;9. 10.3389/fmicb.2018.02854 30542332 PMC6277887

[25] Carvalhaes CG, Cayo R, Visconde MF, Barone T, Frigatto EAM, Okamoto D, et al. Detection of carbapenemase activity directly from blood culture vials using MALDI-TOF MS: a quick answer for the right decision. J Antimicrob Chemother. 2014;69: 2132–2136. 10.1093/jac/dku094 24722840

[26] Tijet N, Boyd D, Patel SN, Mulvey MR, Melano RG. Evaluation of the carba NP test for rapid detection of carbapenemase- producing enterobacteriaceae and Pseudomonas aeruginosa. Antimicrob Agents Chemother. 2013;57: 4578–4580. 10.1128/AAC.00878-13 23817380 PMC3754310

[27] Bernabeu S, Dortet L, Naas T. Evaluation of the β-CARBA^TM^ test, a colorimetric test for the rapid detection of carbapenemase activity in Gram-negative bacilli. J Antimicrob Chemother. 2017;72: 1646–1658. 10.1093/jac/dkx061 28333363

[28] Bakthavatchalam YD, Anandan S, Veeraraghavan B. Laboratory detection and clinical implication of oxacillinase-48 like carbapenemase: The hidden threat. Journal of Global Infectious Diseases. Medknow Publications; 2016. pp. 41–50. 10.4103/0974-777X.176149 27013843 PMC4785756

[29] Burckhardt I, Zimmermann S. Using matrix-assisted laser desorption ionization-time of flight mass spectrometry to detect carbapenem resistance within 1 to 2.5 hours. J Clin Microbiol. 2011;49: 3321–3324. 10.1128/JCM.00287-11 21795515 PMC3165621

[30] Knox J, Palombo E. Performance of a MALDI-TOF MS-based imipenem hydrolysis assay incorporating zinc sulfate. Diagn Microbiol Infect Dis. 2017;87: 258–260. 10.1016/j.diagmicrobio.2016.11.018 27939285

[31] Oviaño M, Barba MJ, Fernández B, Ortega A, Aracil B, Oteo J, et al. Rapid Detection of OXA-48-Producing Enterobacteriaceae by Matrix-Assisted Laser Desorption IonizationTime of Flight Mass Spectrometry. J Clin Microbiol. 2016;54: 754–759. 10.1128/JCM.02496-15 26677247 PMC4767963

[32] Álvarez-Buylla A, Picazo JJ, Culebras E. Optimized method for acinetobacter species carbapenemase detection and identification by matrix-assisted laser desorption ionization-time of flight mass spectrometry. J Clin Microbiol. 2013;51: 1589–1592. 10.1128/JCM.00181-13 23447631 PMC3647957

[33] Pachori P, Gothalwal R, Gandhi P. Emergence of antibiotic resistance Pseudomonas aeruginosa in intensive care unit; a critical review. Genes and Diseases. Chongqing University; 2019. pp. 109–119. 10.1016/j.gendis.2019.04.001 31194018 PMC6545445

[34] Lambert PA. Mechanisms of antibiotic resistance in Pseudomonas aeruginosa. Journal of the Royal Society of Medicine, Supplement. Royal Society of Medicine Press; 2002. pp. 22–26. 12216271 PMC1308633

[35] Potron A, Poirel L, Nordmann P. Emerging broad-spectrum resistance in Pseudomonas aeruginosa and Acinetobacter baumannii: Mechanisms and epidemiology. International Journal of Antimicrobial Agents. Elsevier B.V.; 2015. pp. 568–585. 10.1016/j.ijantimicag.2015.03.001 25857949

[36] Miltgen G, Plésiat P, Mille A, Chatelain P, Fournier D. Detection of carbapenemase activity in Pseudomonas aeruginosa by Matrix-Assisted Laser Desorption Ionization-Time of Flight Mass Spectrometry (MALDI-TOF MS). J Microbiol Methods. 2018;145: 66–68. 10.1016/j.mimet.2017.12.011 29307737

[37] Braud A, Geoffroy V, Hoegy F, Mislin GLA, Schalk IJ. Presence of the siderophores pyoverdine and pyochelin in the extracellular medium reduces toxic metal accumulation in Pseudomonas aeruginosa and increases bacterial metal tolerance. Environ Microbiol Rep. 2010;2: 419–425. 10.1111/j.1758-2229.2009.00126.x 23766115

[38] Carvalhaes CG, Cayô R, Assis DM, Martins ER, Juliano L, Juliano MA, et al. Detection of SPM-1-producing Pseudomonas aeruginosa and class D β-lactamase-producing Acinetobacter baumannii isolates by use of liquid chromatography-mass spectrometry and matrix-assisted laser desorption ionization-time of flight mass spectrometry. J Clin Microbiol. 2013;51: 287–290. 10.1128/JCM.02365-12 23100344 PMC3536220

[39] Chong PM, McCorrister SJ, Unger MS, Boyd DA, Mulvey MR, Westmacott GR. MALDI-TOF MS detection of carbapenemase activity in clinical isolates of Enterobacteriaceae spp., Pseudomonas aeruginosa, and Acinetobacter baumannii compared against the Carba-NP assay. J Microbiol Methods. 2015;111: 21–23. 10.1016/j.mimet.2015.01.024 25644285

[40] Hoyos-Mallecot Y, Cabrera-Alvargonzalez JJ, Miranda-Casas C, Rojo-Martín MD, Liebana-Martos C, Navarro-Marí JM. MALDI-TOF MS, a useful instrument for differentiating metallo- *β* -lactamases in *Enterobacteriaceae* and *Pseudomonas* spp. Lett Appl Microbiol. 2014;58: 325–329. 10.1111/lam.12203 24286119

[41] Johansson Å, Ekelöf J, Giske CG, Sundqvist M. The detection and verification of carbapenemases using ertapenem and Matrix Assisted Laser Desorption Ionization-Time of Flight. BMC Microbiol. 2014;14: 89. 10.1186/1471-2180-14-89 24720586 PMC3997439

[42] Oviaño M, Bou G. Imipenem–avibactam: a novel combination for the rapid detection of carbapenemase activity in Enterobacteriaceae and Acinetobacter baumannii by matrix-assisted laser desorption ionization-time of flight mass spectrometry. Diagn Microbiol Infect Dis. 2017;87: 129–132. 10.1016/j.diagmicrobio.2016.10.016 27863949

[43] Oviaño M, Gato E, Bou G. Rapid Detection of KPC-Producing Enterobacterales Susceptible to Imipenem/Relebactam by Using the MALDI-TOF MS MBT STAR-Carba IVD Assay. Front Microbiol. 2020;11. 10.3389/fmicb.2020.00328 32184776 PMC7058919

[44] Bush K, Bradford PA. Interplay between β-lactamases and new β-lactamase inhibitors. Nature Reviews Microbiology. Nature Publishing Group; 2019. pp. 295–306. 10.1038/s41579-019-0159-8 30837684

