# Supplementary material for: Study on carbapenemase-producing bacteria by matrix-assisted laser desorption/ionization approach
Source: PLoS One. 2021 Mar 18;16(3):e0247369. doi: 10.1371/journal.pone.0247369 (PMC7971901; doi:10.1371/journal.pone.0247369)
Supplement: S1 Table — H–first time point with pronounced hydrolysis (positive results); Hmax- time point with maximum hydrolysis achieved. (DOCX) [file pone.0247369.s001.docx]

|  | **H [min]** | | **H_max_ [min]** | |
| --- | --- | --- | --- | --- |
| Strain | **4 McF** | **7McF** | **4 McF** | **7 McF** |
| ***K. oxytoca* 2 (A)** | **3** | **1** | **10** | **1** |
| ***E. coli* 3 (A)** | **1** | **1** | **1** | **1** |
| ***P. aeruginosa* 4 (B)** | **1** | **1** | **4** | **15** |
| ***P. aeruginosa* 5 (B)** | **10** | **1** | **25** | **1** |
| ***K. pneumoniae* 6 (B)** | **1** | **1** | **1** | **5** |
| ***E. coli* 7 (B)** | **1** | **1** | **1** | **1** |
| ***C. freundii* 8 (B)** | **1** | **1** | **10** | **25** |
| ***C. freundii* 9 (B)** | **-** | **4** | **-** | **20** |
| ***Salmonella* sp. 10 (B)** | **1** | **1** | **1** | **2** |
| ***E. kobei* 11 (B)** | **1** | **1** | **20** | **30** |
| ***K. pneumoniae* 12 (B/C)** | **1** | **1** | **1** | **1** |
| ***K. pneumoniae* 13 (D)** | **25** | **1** | **25** | **1** |
| ***S. marcescens* 14 (D)** | **15** | **1** | **30** | **2** |
| ***E. cloacae* 15 (D)** | **10** | **1** | **15** | **1** |
