# Supplementary material for: Study on carbapenemase-producing bacteria by matrix-assisted laser desorption/ionization approach
Source: PLoS One. 2021 Mar 18;16(3):e0247369. doi: 10.1371/journal.pone.0247369 (PMC7971901; doi:10.1371/journal.pone.0247369)
Supplement: S2 Table — ΔlogRQ—differences between the normalized logRQ of the control variants and treated ones. Negative values mean increase compare to control. In case of results recognized as”non-hydrolyzed” in control variants–values are not shown. (DOCX) [file pone.0247369.s002.docx]

|  |  | ∆logRQ |  | ∆logRQ |  | ∆logRQ |
| --- | --- | --- | --- | --- | --- | --- |
| McF | Strain | PBA |  | PBA |  | PBA |
| 1 | ***P. aeruginosa* 4** |  | ***P. aeruginosa* 5** |  | ***K. pneumoniae 6*** | 0 |
| 1.5 |  |  |  | 0.04 |  | -0.21 |
| 2 |  |  |  | 0.06 |  | -0.15 |
| 2.5 |  |  |  | 0 |  | -0.23 |
| 3 |  | -0.05 |  | 0.06 |  | -0.13 |
| 3.5 |  | 0 |  | 0 |  | -0.1 |
| 4 |  | 0.12 |  | 0 |  | 0 |
| 5 |  | 0.04 |  | 0 |  | 0.02 |
| 6 |  | -0.18 |  | 0 |  | 0 |
| 7 |  | -0.2 |  | -0.05 |  | 0 |
| 1 | ***E. coli 7*** | -0.18 | ***C. freundii 8*** |  | ***C. freundii 9*** |  |
| 1.5 |  | -0.12 |  | 0.16 |  |  |
| 2 |  | -0.15 |  | 0.07 |  |  |
| 2.5 |  | -0.19 |  | 0 |  |  |
| 3 |  | -0.12 |  | 0.10 |  |  |
| 3.5 |  | 0 |  | 0 |  |  |
| 4 |  | 0 |  | 0 |  |  |
| 5 |  | 0 |  | 0 |  | 0 |
| 6 |  | 0 |  | 0 |  | 0 |
| 7 |  | 0 |  | 0.14 |  | -0.18 |
| 1 | ***Salmonella sp.* 10** | -0.18 | ***E. kobei* 11** | -0.15 | ***K. pneumoniae* 12** | 0.15 |
| 1.5 |  | 0 |  | -0.18 |  | 0 |
| 2 |  | -0.08 |  | 0 |  | 0 |
| 2.5 |  | 0 |  | 0 |  | 0 |
| 3 |  | 0 |  | -0.07 |  | 0 |
| 3.5 |  | -0.07 |  | 0 |  | -0.13 |
| 4 |  | 0 |  | 0 |  | 0 |
| 5 |  | 0 |  | 0 |  | -0.1 |
| 6 |  | 0 |  | 0.08 |  | 0 |
| 7 |  | 0 |  | 0 |  | 0 |
